# Perceptions of a family-based lifestyle intervention for children with overweight and obesity: a qualitative study on sustainability, self-regulation, and program optimization

**DOI:** 10.1186/s12889-022-13956-6

**Published:** 2022-08-11

**Authors:** Kaila C. Putter, Ben Jackson, Ashleigh L. Thornton, Claire E. Willis, Kong Min Bryce Goh, Mark R. Beauchamp, Nat Benjanuvatra, James A. Dimmock, Timothy Budden

**Affiliations:** 1 Department of Psychology, College of Healthcare Sciences, James Cook University, Townsville, Australia; 2 School of Human Sciences (Exercise and Sport Science), The University of Western, Perth, Australia; 3 Telethon Kids Institute, Perth, WA Australia; 4 Division of Paediatrics, Faculty of Health and Medical Sciences, The University of Western, Perth, Australia; 5 Kids Rehab WA, Perth Children’s Hospital, Nedlands, Australia; 6 Sports & Exercise Science, La Trobe University, Melbourne, Australia; 7 School of Kinesiology, The University of British Columbia, Vancouver, Canada

**Keywords:** Childhood obesity, Family, Motivation, Treatment, Weight loss, Physical activity

## Abstract

**Background:**

Family-based lifestyle interventions (FBLIs) are an important method for treating childhood weight problems. Despite being recognized as an effective intervention method, the optimal structure of these interventions for children’s overweight and obesity has yet to be determined. Our aim was to better understand participants’ (a) implementation of behaviour strategies and long-term outcomes, (b) perceptions regarding the optimal structure of FBLIs, and (c) insights into psychological concepts that may explain the success of these programs.

**Methods:**

Purposive sampling was used to recruit participants. We conducted focus groups as well as one-to-one interviews with parents (*n* = 53) and children (*n* = 50; aged 7–13, M = 9.4 yr, SD = 3.1) three months following their involvement in a 10-week, multi-component, FBLI involving education and activities relating to healthy nutrition, physical activity, and behavior modification. Using an interpretivist approach, a qualitative study design was employed to examine participant experiences.

**Results:**

We identified three higher-order categories: (a) participants’ program experiences and perceptions (b) lifestyle changes post-program, and (c) recommendations for optimizing family-based programs. Themes identified within these categories included (a) support and structure & content, (b) diet and physical activity, and (c) in-program recommendations and post-program recommendations.

**Conclusions:**

We identified several challenges that can impair lasting behavior change (e.g., physical activity participation) following involvement in a FBLI. On optimizing these programs, participants emphasized fun, interactive content, interpersonal support, appropriate educational content, and behavior change techniques. Concepts rooted in motivational theory could help address calls for greater theoretical and mechanistic insight in FBLIs. Findings may support research advancement and assist health professionals to more consistently realize the potential of these interventions.

## Introduction

Childhood overweight and obesity is recognized as a global public health crisis [1]. In Australia, almost one quarter (24.9%) of children aged 5–17 years were classified as overweight or obese in a recent report [2], mirroring statistics observed in other Western countries, including the United States [3] and United Kingdom [4]. The prevalence of overweight and obesity among children is concerning given the adverse short- and long-term effects of excess body weight on physical [5, 6] and mental [7–9] health. Excess weight in children is linked to hypertension, chronic inflammation, and other cardiovascular disease risk factors (e.g., [10, 11]), and overweight children also tend to experience teasing, discrimination, and ostracism by their peers (e.g., [12, 13]). In addition, children with overweight and obesity have been shown to experience low self-esteem [14, 15] and heightened anxiety relating to body-image concerns and interpersonal relationships (e.g., [16]). If a child’s overweight or obesity persists into adulthood, the risks of long-term physical health complications [17, 18] and poor mental health outcomes (e.g., low self-esteem, depressive symptoms; [19]) are heightened. In light of this evidence, the design and implementation of lifestyle interventions that support weight loss among this at-risk group is a critically important public health challenge [20].

Behavioral and lifestyle interventions (i.e., those excluding pharmacotherapeutic, in-patient, and/or surgical approaches; [21, 22]) aimed at tackling children’s overweight and obesity have been developed using varied approaches and with different ‘target’ populations. Broadly, these approaches may be classified as being child-focused [23, 24], parent-/guardian-focused (e.g., [25, 26, 27]), or family-focused [28]. Despite positive results from some child- (e.g., [23, 24]) and parent-focused (e.g., [26, 27]) interventions, and some research suggesting that parents as exclusive agents of an intervention are as effective as a parent–child dyadic approach in younger age groups [25, 28, 29], it has been acknowledged that interventions provided to families (i.e., targeting parents and children) may have the greatest likelihood of stimulating adaptive short- and long-term weight outcomes for children (e.g., [30, 31, 32]). Lifestyle interventions with families often include resources and support directed toward parent and child nutritional education, physical activity, and behavioral modification (e.g., [33, 34]). As just one example of such a program, the ‘MEND’ (Mind, Exercise, Nutrition, Do it) trial was a prominent UK family- and community-based intervention in which parents and children attended 18 two-hour educational and physical activity sessions (twice weekly) [35]. Following participation in the program, parents were better able to support their children’s dietary needs, and 12-month follow-up tests demonstrated improvements in indicators of cardiovascular health, and psychological well-being among children (evidenced by improvements in global self-esteem scores) [35]. Furthermore, FBLI attendees have reported less concern with weight and shape, a reduction in loss of control of eating, decreased depression and anxiety symptomology, and improved quality of life (physical, emotional, social, and school functioning) at 12 months [36].

There exists a vast literature on frameworks and psychological constructs that may explain varied effects of FBLI programs. For example, in self-efficacy theory, there are four antecedents to an individual’s confidence in their ability to perform a behaviour, namely, performance accomplishments (whether a task is performed successfully), vicarious experiences (whether an individual of equal ability is seen to perform a task successfully), verbal persuasion (i.e., behavioural feedback) and physiological and affective states (such as stress or joy) [37, 38]. In the context of overweight and obesity interventions, positive moods, setting goals and observing parents and peers successfully perform tasks are among the experiences which foster children’s self-efficacy in their ability to make healthy choices [38]. Moreover, the application of self-determination theory to weight loss is not new [39]. According to Ryan and Deci [40], humans have a universal desire to feel a sense of autonomy (i.e., a sense of volition), competence (i.e., a sense that one is capable), and relatedness (i.e., close, supportive connections with others) in their pursuits. In instances when environments, programs, and/or instructors support and satisfy these needs, individuals tend to endorse more autonomous motivation for the activity in question. Self-determination theorists propose that motivation should be understood not only in terms of its quantity (or level), but also its quality (or type). As such, individuals who endorse ‘autonomous’ motives (e.g., enjoyment, interest, value) for an activity or program are more likely to sustain their efforts in the long-term than those who endorse more ‘controlled’ motivation (e.g., reward, incentive, coercion, guilt). Our goal was to explore the types of psychological constructs that participants conveyed were influential in their behaviors and outcomes.”

In discussing the merits of FBLIs, Berge and Everts [30] noted that targeting the whole family—rather than individuals within families—may stimulate more positive weight-related outcomes for children. Similarly, in their review of childhood weight interventions, Latzer et al. [21] concluded that, despite equivocal evidence for some treatment modalities, “the one point for which there is an almost unanimous consensus is the key role of the family in general, and of the parents in particular, to improve the outcome of any treatment program in pediatric overweight” (p. 418). In light of this literature, family-based approaches to tackling children’s weight issues are considered a preferred lifestyle- or behavioral-focused approach for effective weight regulation (e.g., [41]) and for promoting beneficial psychosocial outcomes in children (e.g., [33, 42]).

Notwithstanding the positive outcomes for which family-based treatments may be responsible, however, there remain important questions to be addressed in this area. In a systematic review exploring the viewpoints of adolescents (12–17 years) who had attended overweight and obesity interventions, several of the key themes which emerged were: intervention tailored to the individual, active engagement and fun, longer term support, value derived from professional, peer and family support, and an emphasis on enjoyment from physical activity [43]. Furthermore, the adolescents indicated that the desire for social desirability and improved body image were powerful motivators for weight loss [43]. Considering the range of psychological constructs (e.g., [44]) which may influence participants’ outcomes following the attendance of a FBLI, we were interested in exploring participants’ perceptions of, and the experiences which conveyed, these psychological mechanisms. Thus, questions that were most salient for the design of this study related to the need to better understand (a) the implementation of behaviour strategies (such as self-monitoring) and long-term outcomes, which are notoriously variable in these programs (e.g., [45]); (b) perceptions regarding the optimal structure of FBLIs, and (c) insights into the psychological mechanisms that may explain the success of these programs.

In this study, we sought to understand (aspects of) participant experiences during and following a FBLI for children’s overweight and obesity. In seeking to address the broad issues outlined above, we sought detailed feedback from parents and their children after they had completed a FBLI. More specifically, we sought information regarding participants’ (a) experiences in and perceptions of the program, (b) lifestyle changes following the program, and (c) recommendations for optimizing the effectiveness of programs of this kind. These three broad discussion areas were selected because they either mapped directly onto issues that we sought to better understand (e.g., asking about program experiences and recommendations should inform our knowledge about optimal intervention structure), or because they would likely enable us to make inferences about issues that may be considered too esoteric (or technical) to discuss directly with community-based participants (e.g., behaviour strategies or psychological mechanisms).

## Method

Grounded in our desire to capture parents’ and children’s lived experiences within a family-based intervention, we adopted an interpretivist approach to enquiry [46]. The interpretivist approach is underpinned by the position that multiple realities exist and that these realities are constructed through human interaction. An important tenet of this approach is that knowledge is subjective and socially constructed. This approach, therefore, is suited to underpinning investigations into the ‘realities’ of individuals’ experiences within an activity or context [47]. A qualitative study design was adopted in accordance with our research aims and underlying epistemological stance and theoretical concepts, such as self-determination theory [39, 40], informed the development of the interview guide. Analyses were conducted using Braun and Clarke’s steps of reflexive thematic analysis [48, 49].

### Participants

We aimed to recruit parents and their children who had completed a program and had returned to an extended period of ‘independent’ (i.e., unsupervised) living so as to enable us to explore long-term effectiveness and lifestyle change/maintenance post-program. We primarily sought to obtain insight from parents and children who had received the full program ‘dose’, as those participants would likely have the richest experience upon which to reflect. For the sake of contrast and in order to capture more balanced data, however, we aimed to supplement these accounts with information from a sub-sample of parents who had withdrawn from the program (given that attrition from FBLIs is common; e.g., 50). A total of 53 parents (47 mothers, 6 fathers) and 50 of their children (26 female, 24 male; aged 7–13, M = 9.4 yr, SD = 3.1) participated in focus group interviews (*n* = 97) or one-to-one interviews (*n* = 6 parents). All children were classified, according to body mass index (BMI), as overweight (BMI > 25.0) or obese (BMI > 30.0) at entry into the program, and participants were drawn from the Perth metropolitan area.

### Ethics

Ethical approval for the study was granted by the Human Research Ethics committee at the second author’s university institution.

### Procedures and data collection

All program completers were invited to participate in the study. During a scheduled program follow-up assessment that took place three months after the end of a family’s involvement in the 10-week family-based program (see following section for program information), prospective participants (parents/guardians, and children aged 7–13) were approached by members of the research team (over 50% of those invited were willing to participate). Focus groups were used to provide an in-depth understanding of participant perception and identify barriers to change that cannot be addressed using quantitative methods [43] in a time- and cost-effective manner, as well as examine the role of motivation using a qualitative approach (i.e., gain insights into types, rather than levels, of motivation) [39]. Prior to all interviews, parents were provided with information about the nature of the project and their participation rights, and were subsequently invited to provide written informed consent for their, and their child’s, involvement in the investigation (children also provided assent before participating). Eighteen semi-structured, face-to-face focus group interviews took place at the end of scheduled follow-up sessions in a large, confined, recreational venue that had been use for program activities, with two-to-six participants in each focus group interview. Focus groups for program ‘completers’ were conducted with children (*n* = 9 sessions) and parents (*n* = 9 sessions) separately to ensure that the perspectives of children were freely explored. Six of the parent participants were those who had left the program early, and these parents were separately recruited (via email) in order to provide perspectives from program non-completers. One-on-one phone interviews were held with these non-completer parents to obtain the richest possible accounts from all of these participants.

It is worth noting that some inter-patient and patient-interviewer dynamics likely affected the findings of this current study. For instance, prior to participation in interviews, participants became acquainted through participation in the FBLI program. There is little consensus in the literature on the impact of conducting focus groups with acquaintances. Some researchers (e.g., [50]), suggest that focus groups with acquaintances, particularly children, increases participants’ comfort, facilitating open, honest discussion: whereas others (e.g., [51]) caution against this, identifying an inhibitory effect in this context. In the present context, the use of focus groups was considered an appropriate method of exploring the shared experiences of participants in a non-threatening environment [50]. Further, patient-interviewer dynamics affect findings. In the present study, the interviewers and broader research team were not involved in the implementation of the program and had no prior relationship with program participants. However, all interviewers had prior experience with overweight and obesity research and interventions.

Three members of the research team developed a semi-structured interview guide for parents and children prior to data collection. In line with the aims of this study, the interview guide included open-ended questions developed to explore parent and child (a) experiences within and perceptions of the program, (b) lifestyle changes during and following the program, and (c) recommendations for optimizing the effectiveness of programs of this kind. Probing questions were used to explore answers given by participants, concepts surrounding answers, and to gain further explanation regarding a belief or behavior. At the end of each focus group or phone interview, participants were invited to ask questions. Based on the nature of thematic analysis, there always remains an ability to generate alternate meanings from data, and as a result, data analysis may never truly be considered ‘complete’. Data collection continued, therefore, until members of the research team made an interpretive judgement that the data provided a faithful and detailed representation of participants’ experiences during and following the program [52]. On average, focus group interviews with ‘completer’ parents lasted 22 min, and focus group interviews with children lasted 17 min. The six one-to-one interviews with ‘non-completer’ parents lasted 20 min on average.

### Program description

Accessing a sufficiently large community-based sample of parents and (overweight or obese) children who have attended and completed—and are willing to share their experiences about—a FBLI is a challenging undertaking. For that reason, rather than relying on convenience or snowball sampling, the research team sought to identify a program that could be targeted for the recruitment of all participants, and that could be considered representative—in light of program content and delivery methods—of best-practice in terms of treating children’s overweight and obesity. The Better Health Company (www.betterhealthcompany.org/) provides a childhood overweight and obesity intervention called the Better Health Program (betterhealthprogram.org/) to families across Australia who, over the past 10 years, have shown statistically significant improvements in self-esteem, BMI, healthy eating habits and physical activity upon program completion. The program aims to have children build confidence, improve self-esteem, become fit, and teach parents the skills to ensure their children are healthy. The organization is reputable, well-known, and government funded; its program is based on best-practice programming guidelines and published research evidence (e.g., the previously-described UK MEND trial). The 10-week multi-component program involves weekly, two-hour parent-and-child sessions—delivered by trained health professionals—focused on the provision of resources, education, and experiential activities relating to healthy nutrition, physical activity, and behaviour modification (through, for example, goal setting, planning, reinforcement, and stimulus control; 35). The program targets children with overweight and obesity aged 7 to 13 years old, is cost-free for participants, and operates in venues such as leisure and recreational centers, community and school halls, and council facilities across multiple locations in Australia. Participants are recruited through community promotion (flyers, posters, social media sites) and referrals by health professionals. The Better Health Program—in terms of lifestyle modification aspects, behavior change content, expert delivery, and program length—adheres to many of the best-practice recommendations for family-based program content [20, 53, 54]. As a result, we recruited all participants from this program for the present study.

### Data analysis

All focus groups and phone interviews were transcribed verbatim by the second author and were reviewed a second time to ensure the accuracy of transcription. We conducted reflexive thematic analysis using the six steps outlined by Braun and Clarke [48, 49]. During the first step, the familiarization process, a theme code guide was developed during interviews and the initial transcription process to facilitate the generation of meaning units. Upon completion of the verbatim transcriptions, all transcripts were imported into QSR International NVivo 11.4.2 computer software. During the second step, the fifth author initially identified meaning units (i.e., paragraphs, sentences, and phrases containing contextual information) that had relevance to the research aims. Third, meaning units were isolated and grouped in instances when they contained consistent content (e.g., ideas, experiences). During the fourth step, an initial network of themes was identified that corresponded to the overarching categories that were the focus of discussion.

During the fifth step, the initial network of themes was shared with all co-authors to review. Members of the research team who were not involved in the initial coding adopted the role of ‘critical friends’ in the subsequent analysis stage [47, 55]. In this critical friend stage, the second author met with individuals or groups from the research team (many of whom had extensive experience conducting and publishing qualitative, thematic-analyses-based work) to discuss his initial coding, identification of meaning units, and impression of themes and theme-to-theme relationships. During this process, each of the critical friends offered feedback for consideration, challenged assumptions, provided alternative perspectives, and sought clarification on initial coding. This process resulted in the re-definition, re-organization, merging, and deletion of themes. The goal of this process was not to achieve complete consensus among all members of the research team—instead, the process served to highlight different perspectives and interpretations, and challenge any assumptions or ambiguities. Through the process of analysis, the authors were pushed to challenge their own pre-conceptions. The process of writing and editing the manuscript served as the sixth (and final) stage in clarifying themes and interpretations of the data.

## Results

Themes and subthemes were grouped into higher-order categories according to the discussion areas, and reflected (a) participants’ program experiences and perceptions, (b) lifestyle changes post-program, and (c) recommendations for optimizing family-based programs. We elaborate on these categories in the material that follows, and in the supporting tables we provide additional meaning unit examples to supplement (i.e., different from) those presented in the main text.

### Program experiences and perceptions

Meaning units in this category were grouped into two themes according to whether participants discussed their experiences of support from others in the program (i.e., the human-to-human interactions in the program), or whether they were describing issues relating to program structure and content (see also Table 1). Two subthemes: similarity to peers, and instructor characteristics, were identified within the theme, support. Three subthemes: interactive components to develop competence, novel educational resources, and variety and a sense of autonomy, were identified within the theme, structure and content.

**Table 1:** Themes and exemplar meaning units reflecting perceptions about the family-based program

Theme	Exemplar Meaning Unit
*Support*	“A lot of the kids that are there, are there for the same reasons. My child was able to understand what they’re all going through, so he was doing the same stuff, like eating habits, exercises, stuff like that, and it just gave them something to do together.” (*peer-to-peer support*) “I made a lot of new friends here. It was really fun” (*peer-to-peer support*) (child) “The children were happy. The instructors were supportive… they also take your concerns and help you along. The information was great—for the children to hear it from the group leaders, I think it hits home a lot better.” (*top-down support*) “I feel like it’ll be better to know if we were doing well, so, like, if they kept telling us if we were doing well or all that, then it’ll make us more motivated.” (*desired top-down support*) (child) “Those instructors were great, they supported us every time, they are so patient for the kids.” (*top-down support*) “I think the teachers were really good. They tried really hard to keep the kids focused.” (*top-down support*)
*Structure & Content*	“what I really liked was that there was so much visual stuff. They did this whole experiment, like how much fat is in M&M’s… I hardly ever eat M&M’s anymore. It’s all the visual stuff for the kids, it’s just brilliant. Rather than just saying you shouldn’t eat this, it was just really visual and it was awesome. Sugar was put in your faces to see how much fat was in that product, that was visual. It was a good thing.” (*practical, interactive elements*) “We learned. We learned to eat food in moderation, to check and know how much fat and sodium and stuff’s in the food that we buy from the shops.” (*appropriate educational material*) “We did learn so much about food, what’s good, what’s not, there was lots of information. Finding out stuff that I didn’t expect to find out, that stuff, the food had extra sugar that was hidden and stuff like that, so I found it really good.” (*appropriate educational material*) (child) “If he had a choice to join in [with the physical activity], he would have enjoyed it more, not like it’s a case of ‘you have to do that.’ I felt like it was a little bit forced upon him.” (*enjoyment*)

#### Support

Parents described the importance of a supportive atmosphere in FBLIs of this kind. Importantly, this theme included both ‘peer-to-peer’ support from other program participants, as well as ‘top-down’ support from program instructors.

#### Similarity to peers

In terms of peer-to-peer support, it appeared that high-quality peer support was facilitated in part by perceptions of similarity (in terms of activity and ability levels) between participants. As one parent noted, “[my daughter] finds herself in the middle of a group where they all have the same level of activity, so nobody is faster or stronger or better. That really helped her because, as a group, they all helped together through the same activities.” On a separate issue, another parent appreciated the supportive comments from peers, stating, “saying nice things about other [children] in the group, it builds other people up as well. I think that was helpful, and [the children] were quite proud of it, it’s something for them to hold on to” (see also Table 1).

#### Instructor characteristics

With respect to top-down support from program staff and instructors, parents acknowledged the significant role played by instructors in terms of setting the tone for support in the program. It was important that instructors in programs of this kind were seen as friendly, credible sources of information, and displayed empathy and patience. One parent commented, for example, “I would love to thank [instructor 1] and [instructor 2]. It was great, they were great, we had a really good time, they supported us well because they were so patient with the kids.” In a similar vein, another parent commented on the positive effects engendered by instructors creating a sense of ‘groupness’, noting, “I found it really interesting. [Our instructor] was really good, we had lots of interaction, a lot of group things we had to do, a lot of group discussions among parents.” Complementing these experiences was feedback from participants who had failed to complete the program, and who also considered support-related issues to be key to program effectiveness. One parent commented, for example, “I work full time, I have two kids, my husband works night shifts—it’s like I'm a single parent, so I'm really looking for support from [the instructors]. Sometimes, it felt like they more cared about filling in forms than being of support to anyone. I didn't feel like it was something we were going to get supported through.”

#### Structure & content

In this theme, parents and children described several structural and content elements of family-based programs. Most notably, they emphasized (a) the significance of practical and interactive components, (b) the inclusion of appropriate educational material, and (c) the importance of program features that support participant enjoyment.

#### Interactive components to develop competence

With respect to practical and interactive components, parents and children appreciated the ‘hands-on’ elements of program content. This practical approach allowed parents and children to develop their understanding (and competence) regarding, for example, nutrition-related skills. Specific strategies will vary program-by-program, but one example of this interactive approach in the present program was the use of food label reading sessions, and a ‘live’ visit to a supermarket. Discussing this component, one parent commented, “the best thing we did was when they took us to the supermarket and they actually showed us how to look for the labels… like, telling us ‘this is good, this is not good, this has too much fat, this one has a little bit of sugar’, it just gives us details on what to take away.” Children also appreciated these practical methods for delivering educational content. One child commented, “I liked it when we went to the shop and learned about the labels. We got to learn about the labels and what’s good for you, what’s bad for you, and what the best brands are.” Similarly, reflecting on ways in which program content could be improved, another child noted he would have appreciated “if we got to learn things in different ways, like doing activities, instead of sitting and… trying to listen.”

#### Novel educational resources

As well as describing the importance of practical program elements, participants also expressed a desire for educational material—which was central to program content—that was considered ‘new’ or ‘fresh’. For example, the program afforded parents and children with the opportunity to learn new healthy recipes; one parent noted that “[the] program’s been really helpful in terms of recipe ideas and giving information about reading labels and all those kinds of things I didn’t know before.” Similarly, another parent commented, “A lot of the stuff… was all new for the kids, and the information was very accurate. They kind of gave you websites that you can look for, go and search for recipes, community activities and things like that.” It was noteworthy that children expressed the same sentiment. One child commented, “I learned that when you have lots of muscle, fat won’t be able to grow that much. And also, instead of using sugar, you can use natural sweeteners or healthy fruits. And instead of having white bread, you could have whole-meal bread. I found out that wheat is much better.” Whilst discussing educational material, parents expressed concerns when information was not considered to be ‘new’ or ‘fresh’. One parent, for instance, noted that “I think the program itself is good, but I don't think it was suited to my son. You know, they gave a lot of good tips and things, but it was nothing that wasn't already common knowledge.” A parent who did not complete the program added, “it’s all good to say ‘you need to stay away from junk food’, but we all know that already. It was based a lot on things that we already know… we stopped going because everything that we were learning was stuff that everybody knows.”

#### Variety and a sense of autonomy

Finally, participants emphasized the importance of program elements that support participant enjoyment. Many parents and children reported that they enjoyed the physical activity within the program sessions. One parent summarized, “the exercise, that’s the fun part. Because they’re all the same, they helped each other, and they had real fun, they loved it. The kids will go off with the instructors and get bright red, sweaty, and they absolutely loved it, and they would stay behind”. This enjoyment appeared to be derived from children being able to access a variety of different forms of physical activity, and ‘having a say’ (or some degree of autonomy) over their activity involvement and choices. Participation in different team or cooperative activities was perceived as enjoyable and as an opportunity to learn and develop competence in new areas. One child noted, “I liked the sports. We got together and worked as a team. And we got to run around and be free, have fun, and learn new stuff. Bouncing! And running! I liked getting to do the different exercises.” The same effect was described in instances when children felt that there was a lack of variety or autonomy. One child commented, for example, “I didn’t like having to do fitness, it didn’t really help me. We were just standing in circles for the whole thing, we didn’t run or anything. I thought we’d be racing and that stuff.” One parent echoed this sentiment, noting, “[my boy] kind of felt like it was a bit, like he had no choice with that side of it, that’s how he felt, that's what he said to me” (see also Table 1). Another parent elaborated, “[My child] told me that if it’s boring they don't want to go, so I feel that the activity part could attract them to go. Something that attracts them and not something simple, and then they would do it. It would be good if there were some interesting activity for them.”

### Adherence to lifestyle changes post-program

When describing their ability to maintain lifestyle changes following a family-based weight loss program (i.e., to self-regulate in the longer-term), participants spoke broadly about two key health behaviors. Specifically, they described successes and challenges relating first to children’s diet, and second to children’s physical activity levels, which were grouped into two themes. In doing so, participants also described some of the reasons underlying those successes and challenges. In the material that follows (and Table 2), we detail instances where families felt that they had successfully sustained or self-regulated their diet or physical activity following the program (i.e., ‘successes’), in addition to those instances where they felt they had failed to sustain or self-regulate those behaviors as well as hoped (i.e., ‘challenges’). Within the theme, diet, these responses were grouped into three subthemes, namely: healthier dietary choices, resources and goal setting, and conflicting priorities and financial cost. Four subthemes: habit formation, lack of motivation and structure, stigma and ostracism, and financial constraints, were identified within the theme, physical activity.

**Table 2:** Themes and exemplar meaning units reflecting lifestyle (i.e., dietary and physical activity) adherence post-program

Theme	Exemplar Meaning Unit
*Diet (Success)*	“The way I cook now, I reduce the amount of oil in cooking, reduce what they call it, the portion or the size.” (*generally healthier choices*) “I used to bring full cream milk, you know, for the small kids. Now I’m just bringing low fat. I used to bring white bread and whole meal, now I’m bringing the whole meal only. I don’t bring white bread at all.” “He still has snacks but now, he has different snacks, like fruit salad with yogurt, and he likes it.” “They are looking for more vegetables instead now, and eating more vegetables now.” “I started drinking a lot more water instead of lemonade.” “I know I always feel hungry at night, so I now normally eat apples. Before that I always had cookies but now I don’t.” (*healthier substitutions*) “If I like see a packet of chips, I turn it around, and then see the nutrition label. For chips, I look at the salt, and then for like lollies, it’s like sugar, I really just look for everything, cause they’re full of everything. (*nutrition resources, tip, and skills*) (child) “Things like how to cut down on carbs, and we learnt how to read the labels, and even still to this day, they still do it. With my son, he’s become very observant. When we go shopping he’s like, “Ma, this is not good, it’s got that much sugar”. He’s just more conscious now, and he goes through the labels.” (*nutrition resources, tips, and skills*)
*Diet (Challenges)*	“We made a bit of a WhatsApp group for sharing ideas, which we haven’t actually connected, we were going to, but I suppose everyone got busy. We have got a Facebook group now, but we haven’t really talked during this holiday, probably cause everyone’s away or something.” (*conflicting priorities*)
*Physical Activity (Success)*	“The program gave us these pamphlets with different things you could do. Crossfit was one of them. So, I’m doing Crossfit once a week now.” “I was allowed to ride to school afterwards, and at first, I took the short way, but now, I always go the long way.” (*continued positive attitudes and engagement*)
*Physical Activity (Challenges)*	“I think it’s [physical activity] dropped off, because in the program we were setting goals, like their physical activity goal every week. It was set up from the start and you had to do it for about a month, so they were pushing. Now that’s gone, my son doesn’t do that anymore.” (*loss of structure*) “The problem is like, we give excuses, I’m busy, I’ve got something, I can’t do it today, today I’m tired, always making excuses.” (*loss of structure*) “Like mixed martial arts, self-defence physical activity, you do your own stuff, and you never get kids get left out. They do stuff together, but no kids get left out because they’re the slowest or fattest or shortest or tallest. That worked for us, we don’t feel like he’s getting left out, where in soccer and stuff they get left out. It’s about finding something that they want to do, and enjoy, get accepted. If groups were more inclusive, that would be good.” (*stigma / exclusion in community activity*) “Some of the community sports are too expensive. We have 3 kids, and it’s too expensive to put them in there.” (*cost*)

#### Diet

Three months following the completion of their program, parents and children described various ways through which they (believed that they) had successfully adhered to, or sustained, dietary changes brought about during the program. These ‘successes’ reflected an ongoing awareness—that had been provided through participation in the program—regarding (a) healthier choices, recipes and food substitutions, and (b) nutrition resources (e.g., websites) and simple ‘tips’ or skills (e.g., portion size changes, label reading habits) that were considered valuable and sustainable.

#### Healthier dietary choices

Speaking generally about making healthier dietary choices, one parent noticed, “we’re now eating more fruits and vegetables. We don’t have food goals, but we’re just eating more of the healthier stuff now.” Similarly, one child mentioned, “we make these cupcakes now, and we didn’t put any sugar on them at all. We do it healthier, the healthier version.” Another child highlighted that his family was still making healthier substitutions that had been introduced during the program, “now we use wholegrain bread all the time. And also wholegrain spaghetti, and wholegrain crackers” (see Table 2).

#### Resources and goal setting

Participants also described making sustained use of food-related resources and some of the ‘simple’ skills that they had picked up during the program. Online resources, for example, were used by families to source and prepare healthy meal ideas. One parent noted, “there’s a website which gives you 4 meals every week, and it shows you how to prepare… some of us have taken up that, so we’re still getting 4 healthy meals a week.” In terms of the tips and skills that had been maintained, participants most frequently identified issues relating to label reading and portion control, which had been core elements of the program. One parent commented, “one thing we’ve been able to continue is a lot of the label reading—understanding how to read labels on food. You know, we still do that all the time—look at how much sugar, how much fat, so at least we don’t eat a lot of that sort of stuff now.” Another parent reinforced this aspect, indicating, “he’s more aware now of food labels and, you know, the portions. So now when we go shopping, he will still look at the labels for the salt and sugar, and the fats. He didn’t do that before, but he does that now.” Children also highlighted the continued use of this information. One child mentioned, for example, “I’ve changed my eating habits, and when we shop, I always read and check the labels.” In addition to label reading, parents also noted they had sustained a focus on portion sizes following the program. One commented, “[my children] are always watch their serving sizes now. They tend to make it smaller now.” Another parent described the ongoing use of goal-setting principles, initiated during the program, in relation to portion control, “my daughter set goals. One of her main goals was not to ask for second servings. She used to throw tantrums in the past, but now she’s better.”

#### Conflicting priorities and financial costs

As indicated above, there were multiple ways in which parents and children had sustained dietary changes following the completion of the program. There were, however, important challenges to maintaining healthy dietary practices once families were no longer attending the program. These challenges appeared to reflect issues that were related to conflicting priorities or financial cost. In terms of conflicting priorities, one parent described difficulties with their child’s preference for hedonically rewarding food options, saying, “I can’t see him ever choosing an apple or a carrot over a piece of chocolate, even though he now knows how bad the chocolate is, and how good the apple or carrot are.” In addition, some parents described how financial pressures contributed to the sense that sustaining healthy food habits was difficult outside of the program. One parent indicated, “I pretty much live day-to-day with what I can and can’t get, and fresh fruit and vegetables are on the ‘luxury’ list. We just can’t afford to get fresh fruit and vegetables every week, so that made it hard for me. [The children] are telling me you should prepare this and this, and then… I can’t afford to buy this and this for them to eat” (see also Table 2).

#### Physical activity

There were several instances where parents and their children described the continuation of more positive attitudes toward physical activity—and in some instances, physical activity levels—after the program.

#### Habit formation

One child commented, “I’ve been walking my dog, walking around, running around, and I’ve been doing sport at school. It was one time, then it went up to two, then three, now it’s four times a week.” Similarly, another child noted that they had continued their involvement in the activities that were enjoyable in the program, “I do lots of sports at school now. Seven hours a week after school, because I like to play soccer now.” Some parents reinforced this lasting change in attitude and behaviour—one parent explained, “We started walking to school during the program, and we’ve continued with that. Not every day, but we do it often—walk to school in the morning, we really enjoy that.” One parent reflected on the continued change in her son’s activity levels, “after the program, like he does ride his bike, I think that gives him motivation to keep going. Now, if he misses his bike ride on Monday, he will do it on Wednesday, or he tries to put it on other days, so that he knows he’s done it [cycled] twice or three times a week.” Another parent added, “[my daughter’s] at home and, ‘Oh, I’ve got some time now, I’m going to go outside and play on the trampoline’, she does that almost every afternoon, so even though it might not be organized activity, she’s always like, ‘I’m going to go run around or go do something’.”

#### Lack of motivation and structure

In comparison to the range of ‘successes’ parents and children described regarding their sustainment of dietary changes, though, maintaining physical activity patterns following involvement in the study’s family-based program appeared more challenging. There were, accordingly, substantial challenges that parents described in relation to the self-management (within families) of physical activity. First, parents highlighted that the withdrawal of the structure provided by the program (and allocated time and supervision) had made it difficult for them to continue to support their child’s physical activity. On this issue, one parent commented, “it’s hard for me—there’s not enough time. Sometimes, we [the parents] don’t have the energy. Sometimes, we have the time, but we get more tired than the children, they have energy, but it’s just us that doesn’t.” Another parent explained, “The program’s just really good, it’s just the challenge of having, of how to go on on your own, because it’s very goal oriented, that’s been so good, it is more of how you keep them motivated to carry on.” A different parent reinforced the same issue, “The exercise part for the kids is missing after you finish, so we had to fill the time, even if he does other things, it’s not the same, because if you’ve got something interesting, they get more into it I find. I think just not having the fixed times, that we knew that Thursdays four to six, that’s what we’re going to do.”

#### Stigma and ostracism

In reflecting on the benefits of being with similar others during the program (e.g., other overweight children), some parents noted that integration into ‘regular’ community activity post-program was difficult. One parent explained that her child felt ostracized when she tried to participate in community-based sport following the program, “the people organising the activities don’t use your kids in games because they’re “fat” or they’re “slow”, and it’s adults saying that to kids.” Another parent shared, “when [my son] was doing the exercises, he was actually so confident and relaxed because everyone else was the same… it’s the interaction with other kids. It’s just different after [the program] when they go out and to do the sports on their own” (see also Table 2).

#### Financial constraints

Finally, in the same way that financial pressures were highlighted as a challenge to ongoing dietary changes, the cost of accessing courses, gyms, or social sports was another challenge to maintaining physical activity initiated during the program. One parent noted, “even the courses… to join them it’s a bit expensive, that’s why sometimes it would be better if it was free somehow. You know, it’s good for us and the kids, so we’d love if there are any more things coming, like for active sport, these kinds of things.”

### Program Recommendations

Meaning units within this category specifically related to parents’ and children’s recommendations for optimizing FBLIs for childhood overweight and obesity. These recommendations either reflected those that were provided about the program experience itself (grouped into the theme, in-program recommendations), or those focused on the period following the program (grouped into the theme, post-program recommendations). Below, we detail key issues highlighted in these two themes (see also Table 3). Within the theme, in-program recommendations, three subthemes: intervention length and a gradual transition, increased physical activity, and identification with peers, were identified. Additionally, three subthemes: ongoing program support, ongoing community support, and accountability, were identified within the theme, post-program recommendations.

**Table 3:** Themes and exemplar meaning units reflecting suggestions for program recommendations

Theme	Exemplar Meaning Unit
*In-program Recommendations*	“Maybe do these programs over a longer period of time, because you always fall off once you’ve finished. So maybe extending them would be good.” (*intervention length*) “The children want to exercise, they want to move, they don’t want to sit down and listen. More activity-based things could be more engaging.” (*intervention content*) “The younger ones are seven to nine, they’ve got a really short attention span, so it was quite long, after a day at school, to come in and do the program. The older ones, sort of, are more aware of the impact of having a weight issue, it’s more obvious to a nine to eleven maybe, or a nine to thirteen.” (*grouping considerations*)
*Post-program Recommendations*	“I think regular height and weight checks once a month, every two months maybe… I think that’s really important for the children and even the parents… and they can talk for ten to twenty minutes about what they’ve been doing different.” (*maintaining connections – program*) “Call and speak to the child… ‘hey, how’re you going, what sport are you doing now, how’s school going?’ And, you might be able… to keep them on track, like a long-term thing after the program.” (*maintaining connections – program*) “After the group finishes, maybe if there was somebody who could still do like the activity side, even just an hour a day or an hour a week. If we could do that for the rest of the year, it’d really get them motivated and going, then maybe that would help.” (*maintaining connections – program*) “It’d be nice to have sessions every now and then, get them back together again. I think, like an email chat, just send the children an email, how you going, that kind of thing.” (*maintaining connections – program*) “I think an app, like most people have smartphones, so an app would certainly keep me on track.” (*maintaining connections – other participants*) “I know HBF do these fitness sessions. How about something like that from the program for the kids in certain areas? I think that would be really great, because then it’ll be engaging for the kids, and they really want to be a part of it. I think something like that would be really good.” (*maintaining connections – program and other participants*)

#### In-program recommendations

When asked how to achieve the best possible short- and long-term outcomes from these kinds of programs, parents and children made recommendations about in-program issues specifically relating to intervention length, intervention content, and grouping considerations.

#### Intervention length and a gradual transition

In light of some difficulties maintaining healthy behaviors (specifically, physical activity) following the program, some parents suggested that longer programs (including a dedicated ‘transition’ phase at the end) may be beneficial. One parent commented, “I think it would be nice if programs were spread out, instead of having it in just one school term.” On the issue of a gradual transition out of the program and into self-regulation, another commented, “it would be nice to have a bit more continuity instead of just doing that one program and finishing.” One parent suggested that a transition phase could involve shorter sessions, “You could, you know, make those later sessions just a quick blast of activity. We’ve got what we need for information, but that could be a way of keeping things active for longer.”

#### Increased physical activity

In terms of intervention content, it was apparent that children expressed a desire for prioritizing time toward (enjoyable) physical activity during the sessions. One child mentioned, “less talking, more playing. We normally do an hour of talking and a bit of sport, and then we’re coming back, and then we have to leave.” Similarly, another commented, “I want more games, I want to run more. If not, we do more mind things instead of active things.”

#### Identification with peers

Finally, some children raised noteworthy recommendations about sensitivity to grouping considerations. Although the majority of children observed likeness with others in terms of weight and health issues, age differences appeared to be a barrier to participation in some instances. One child mentioned, “I don’t know, maybe the age groups that they take in at one time should be smaller, because for me to come in was awkward, because I’m 13 and all of you guys are like 10.” Another child commented, “I kind of wished like the age groups were exactly the same for everyone. You won’t get a lot of teenagers wanting to join unless their parents force them into it.”

#### Post-program recommendations

Parents provided valuable recommendations for post-program strategies that could improve the maintenance of healthy dietary and physical activity behaviors learned during family-based programs of this kind. The overarching recommendation reflected the importance of maintaining connections—with the program and those with whom the families had completed the program.

#### Ongoing program support

For example, parents endorsed the possibility of having formal ‘post-program support’ through occasional contact with the children. One parent explained, “to have someone like that [from the program] calling, you know, contacting you, asking how you’ve been, what you’ve been doing, what your goals are for the next month, I think that would just reinforce the message, and it wouldn’t take a lot of time.” Other parents felt that a continued connection between program and parent would help ensure children were able to maintain their newly-learned healthy behaviours. One parent said, “it’d be a good back-up, like a review or something. Maybe we should make it every 2 or 3 weeks, just to keep us motivated.”

#### Ongoing community support

In addition to formal connections with the program, parents also indicated the benefits of facilitating continued connections with one another. Parents suggested that social media platforms could be used to create a lasting sense of community, allowing them to connect with similar others and organize social and physical activities. One parent summarized, “I think creating a Facebook group would be fantastic. You can connect with everybody together, you and the kids can meet somewhere, they can play in the park or walk around.” Another stated, “we started a thing online through Facebook, we sort of just join our own personal group to keep helping each other, and you know, we can add whatever we want to, and sort of check in on each other to see how we’re going, and that has helped.”

#### Accountability

Finally, one parent suggested utilizing gamification principles in this respect, “Like, a five-day challenge, you can do it on Facebook or Messenger. It comes through, post up what you had for breakfast, get everyone involved. It’s going along the nutrition lines, you’ve got your five days a week included, and another’s posting up all the meals you had in a day, and adults can get into that sort of thing, I think it’s a great idea to actually get people on track” (see also Table 3).

## Discussion

Lifestyle modification interventions are an important strategy for treating childhood weight problems, and family-based programs delivered to parents and children are among the most effective approaches for delivering such interventions to children and pre-adolescent youths (e.g., 5–11 years, 32; ≤ 10 years, [45]). There remain, however, important issues to be addressed in order to enable health professionals to effectively and consistently realize the health promotion potential of these interventions. Three of these issues formed the basis for the present study. First, little is known about long-term outcomes and effective self-regulation of participants following program completion (e.g., [45]). Second, there is a need to better understand perceptions about the optimal structure (e.g., program content, delivery, modality, length) of FBLIs. Third, more insight is needed into the psychological mechanisms and theories that may help researchers and health professionals better understand the success of family-based programs of this kind.

### Long-term outcomes and self-regulation

Family-based programs of this kind are developed with the aim of modifying lifestyle behaviors during and following the intervention; two particularly prominent behaviors in this respect are physical activity and diet (e.g., [56]). Based on the experiences that participants shared in this study, it appeared that long-term success was more feasible with respect to aspects of dietary (rather than physical activity) change. Simple dietary skills and behaviors, including reading food labels, choosing healthier recipes and substitutions, using nutrition resources, and adhering to portion control, appeared to be habituated following the program for many families. In contrast, although a large proportion of participants described the retention of more positive physical activity attitudes post-program, fewer families had converted this into greater physical activity participation.

Some of the barriers to lasting dietary and physical activity change (post-program) identified in our results such as cost, time, and competing priorities as barriers to physical activity and lifestyle change are well documented and understood (e.g., [57]). However, notably, parents described experiences of exclusion and stigma as they attempted to integrate children into community activities after intervention cessation. The structure of the program, the support of instructors, the sense of ‘groupness’, and the similarity between participants appeared to protect against these challenges during the program. However, post-program participation in community sport and exercise was, in some cases, compromised by ostracism and rejection. Weight-related stigma can be pervasive in all aspects of the lives of children with overweight and obesity [12, 58], and in this context it may contribute to failures in the continuation of physical activity once the support and structure of a program is withdrawn.

From a practical perspective, as well as being mindful of support strategies that are commonly recommended in this area (e.g., subsidizing the cost of ongoing physical activity participation post-program), addressing specific stigma-related physical activity challenges might help programmers translate improvements in physical activity attitudes into sustained physical activity participation. In terms of methods for doing so, participants spoke about the benefits of end-of-program transition periods, and strategies aimed at ‘maintaining connections’ with one another (and with program staff) following the completion of a family-based program. The benefits of these continued connections (e.g., activity groups, social media groups) may not only provide opportunities and social support for physical activity [59], they may also help to ‘insulate’ program participants against stigmatization by providing a ‘safe’ physical activity environment. Indeed, there is recent evidence that exercising alongside other members of (what is considered) a stigmatized population may actually serve to increase adherence and enjoyment for those within the group (e.g., [60]).

### Optimal program design

It is often recommended that family-based programs accommodate themes of nutrition, physical activity, and lifestyle modification (e.g., [20, 53], [54]). Within those broad themes, however, the present findings provided nuanced insight into some of the specific elements and experiences that participants considered important. First, it was noteworthy that participants emphasized enjoyment as a key program experience. Enjoyment appeared to be most commonly derived through the provision of physical activity opportunities, and was maximized when these opportunities were tailored, varied, and allowed for participant input and autonomy. From a nutrition and education perspective, it also appeared that participants were able to discern material that was new and fresh (i.e., interesting) from that which was deemed uninformative. These findings underscore the importance of family-based programs providing contemporary, evidence-informed information (ideally) in practical and interactive ways. We reflect on the theoretical underpinnings of these findings in the following section; from a practical standpoint though, health professionals are encouraged to adhere to these recommendations in seeking to create enjoyable and stimulating experiences within family-based programs. Finally, with respect to program structure, although many programs incorporate behavior change principles—such as self-monitoring, goal setting, and planning—within intervention content [56], participant responses in this investigation emphasized the importance of continuing this support following the conclusion of the ‘main’ program stage. Parents, for example, commented on the benefits of a gradual, supported transition following the main program stage, and a maintained connection with programmers and peers. These suggestions incorporated many of the behavior change principles that are known to be effective in supporting successful lifestyle change (e.g., planning, feedback, self-monitoring, social support, reward, repetition, and habit formation e.g., [61]).

### Psychological mechanisms

Several of the issues identified by participants mapped onto elements of motivation theory, and in particular, self-determination theory [62]. It was noteworthy in the present study that positive experiences of physical activity were those described as fun and enjoyable (i.e., autonomous motives), and that positive feelings about the educational content in the program were attributed to the material being interesting or valuable (i.e., other autonomous motives). Given that there is some evidence of links between autonomous motivation in weight loss interventions and longer-term weight outcomes (e.g., [63]), assessment in family-based programs might be targeted more consistently at studying trajectories in, transfer between, and outcomes of, parent and child motivation.

An additional element of self-determination theory with relevance to these findings is the notion of basic psychological needs [40]. It was particularly interesting, therefore, that without being explicitly asked to describe these needs, the concepts of autonomy, competence and relatedness were apparent in participants’ descriptions about their involvement in the program. Perhaps most notably, we observed multiple instances where participants discussed strategies that supported (or that would support) relatedness. To illustrate, participants (a) appreciated the (top-down and peer-to-peer) supportive atmosphere in the program, (b) were reassured by their similarity to other participants, (c) presented considerations about appropriate grouping strategies in these programs, (d) enjoyed cooperative sporting activities, and (e) desired strategies to support the continuation of bonds formed during the program.

Aside from relatedness issues, we also obtained feedback relating to the importance of autonomy and competence development (e.g., comments on exercise preferences and program content), and regarding the importance of variety, which has also been shown to predict autonomous motivation [64]. It is possible, therefore, that a more systematic application of self-determination (and other motivation) theory in this context might benefit researchers and program developers alike. For researchers, self-determination theory might provide opportunities to demonstrate how parents support (or thwart) their child’s autonomous motivation during and following a program of this kind, or to study how family motivation variables shape intervention outcomes. Meanwhile, for health promotion practitioners, this framework might inform how to structure intervention content and delivery such that participants’ psychological needs are met.

### Limitations

There are many factors—such as socioeconomic issues (e.g., educational level, access, socioeconomic status, family structure; e.g., [65, 66, 67]), that may drive or interact with families’ capacities to self-regulate and achieve long-term outcomes from these programs. Data was not collected nor were analyses stratified to include these factors in this qualitative study. Future research may benefit from explicit consideration of these variables. It is important, for example, to use similar methodologies in further work in order to better understand, in their own words, children’s experiences of physical activity-related stigma and ostracism following their participation in family-based programs. In doing so, researchers may also be able to identify and test suitable methods for intervention that circumvent these problems. Additionally, the average length of the children’s focus group interviews was 17-min, which may have resulted, in part, from the memory constraints and attention spans associated with child respondents [68]. Interview length recommendations for focus groups involving children vary substantially (20–120-min), and some recommend a break between sessions [50]. Furthermore, a closer consideration of age differences, participants’ level of familiarity, and gender differences (among participants and the interviewer) during group allocation might elicit more fruitful interactions in future research [50]. Similarly, parent focus groups and one-on-one interviews lasted approximately 20–22-min. While the concept of data saturation guided the authors’ qualitative research approach, it is acknowledged that the notion of saturation is contentious [69]. The authors do not claim to have ‘finalized’ the experiences of FBLI attendees and acknowledge that, by being reflexive about saturation, conducting longer or multiple interviews may have elicited novel findings [52, 70]. While the individual phone interviews were conducted to accommodate non-completers, rapport can be more difficult to establish during phone interviews and they can be more demanding and fatiguing and thus tend to be shorter [71]. Moreover, focus groups contained 2–6 participants, however, while recommendations vary, some research suggests larger focus groups are preferable [72]. It is also important to acknowledge that the experiences we captured were from participants within a single family-based program. As such, we note that there may be idiosyncratic accounts within these findings that do not represent those of other populations. Furthermore, we were unable to report the exact number of invitations distributed and acknowledge that study participants may not be representative of all attendees of the Better Health Program.

## Conclusions

To mitigate weight-related stigma impacting long-term change, families emphasized end-of-program transition periods and strategies to maintain connections with both program staff and participants. On optimizing the program design, participants highlighted a desire for autonomy in program activities, tailored, interactive and novel content, and their similarity to other participants. In line with self-determination theory, participants’ desire for autonomy, competence and relatedness suggested they were driven by autonomous motivation, which has the potential to bolster long-term outcomes. Other barriers to lasting behavioural change post-program included time and financial constraints and competing priorities. The focal program in this study is empirically grounded, accommodates many best-practice recommendations, and consists of elements common across programs of this kind. That being the case, it is reasonable to expect that the evidence reported in this investigation may be informative for other programs and researchers [73]. It would be interesting in the future to examine the extent to which these findings do transfer to other programs and settings. Similarly, although we accommodated a three-month ‘self-regulation’ (post-program) window, it would be fascinating to compare reports at that time point with discussions immediately upon conclusion of the program and those collected some time later (e.g., one-year post-program).

## Data Availability

The datasets generated and/or analysed during the current study are not publicly available due to the nature of the data but redacted transcripts are available from the corresponding author on reasonable request.
